# Seawater Membrane Distillation Coupled with Alkaline Water Electrolysis for Hydrogen Production: Parameter Influence and Techno-Economic Analysis

**DOI:** 10.3390/membranes15020060

**Published:** 2025-02-11

**Authors:** Xiaonan Xu, Zhijie Zhao, Chunfeng Song, Li Xu, Wen Zhang

**Affiliations:** 1 Tianjin Key Laboratory of Indoor Air Environmental Quality Control, School of Environmental Science and Technology, Tianjin University, Tianjin 300350, China; 2 State Key Laboratory of Chemical Engineering, School of Chemical Engineering and Technology, Tianjin University, Tianjin 300350, China; 3 Tianjin Key Laboratory of Membrane Science & Desalination Technology, School of Chemical Engineering and Technology, Tianjin University, Tianjin 300350, China

**Keywords:** membrane distillation, alkaline water electrolysis, hydrogen production, integration, simulation and optimization, techno-economic analysis

## Abstract

The production of green hydrogen requires renewable electricity and a supply of sustainable water. Due to global water scarcity, using seawater to produce green hydrogen is particularly important in areas where freshwater resources are scarce. This study establishes a system model to simulate and optimize the integrated technology of seawater desalination by membrane distillation and hydrogen production by alkaline water electrolysis. Technical economics is also performed to evaluate the key factors affecting the economic benefits of the coupling system. The results show that an increase in electrolyzer power and energy efficiency will reduce the amount of pure water. An increase in the heat transfer efficiency of the membrane distillation can cause the breaking of water consumption and production equilibrium, requiring a higher electrolyzer power to consume the water produced by membrane distillation. The levelized costs of pure water and hydrogen are US$1.28 per tonne and $1.37/kg H_2_, respectively. The most important factors affecting the production costs of pure water and hydrogen are electrolyzer power and energy efficiency. When the price of hydrogen rises, the project’s revenue increases significantly. The integrated system offers excellent energy efficiency compared to conventional desalination and hydrogen production processes, and advantages in terms of environmental protection and resource conservation.

## 1. Introduction

Green hydrogen is an indispensable raw material in various industrial processes, and a clean medium for the transportation and storage of renewable energy in future sustainable energy systems [1,2]. Alkaline water electrolysis (AWE) is the cheapest industrial electrolytic water technology for producing green hydrogen. However, the cost of green hydrogen produced by AWE is still not competitive with that of grey hydrogen produced from fossil fuels [3,4]. Fortunately, using renewable energy to provide electricity for AWE is expected to significantly reduce the cost of green hydrogen and offer broader opportunities for its large-scale application in the future [5]. In addition to electricity, water electrolysis also requires a sustainable supply of pure water. However, freshwater is a finite resource (<1% of Earth’s water), and there are water shortages worldwide. Therefore, using non-drinking water sources, such as groundwater, sewage, and seawater, to produce green hydrogen is very important, especially when freshwater is scarce [6,7,8].

Seawater accounts for about 96.5% of the earth’s water. Using seawater as a raw material for hydrogen production has great prospects [9,10]. There are two strategies for utilizing seawater to produce hydrogen. One is using seawater as the water source directly, by which strategy the system is more intensive, and equipment costs would theoretically be saved. However, this is accompanied by the problems of corrosion and pollution of electrode materials in high-chlorine environments, and competing reactions of chlorine emissions. These problems make it very difficult to develop suitable catalysts and electrodes, as well as making this a high-cost process. Thus, it is still being studied in the lab [11]. The other strategy is desalinating seawater for use as a water source for water electrolysis. Along with the development of industrial production capacity, desalination technology and pure water electrolysis for hydrogen production are well developed and used in industrial production. Furthermore, the cost of desalination has been reduced to an acceptably low level, based on economic analysis [12]. Research shows that seawater desalination’s energy and cost requirements are below 1% of those of water electrolysis [13,14]. Therefore, compared with direct electrolysis of seawater, indirect seawater electrolysis after desalination has considerable advantages in terms of cost and technical feasibility.

Membrane distillation can use heat as a driving force to produce pure water from seawater [15,16]. Meanwhile, the energy efficiency of AWE is about 70%, and the rest of the electric energy is converted into waste heat. Therefore, membrane distillation technology can be coupled with seawater desalination to produce pure water that supplies the electrolytic water process. This integrated process can simultaneously realize the coupling of material and energy flow [17].

Recently, the SEA2H2 project in Wageningen demonstrated the technical feasibility of producing hydrogen from seawater via an integration technology based on cascading membrane distillation and a proton-exchange membrane water electrolyzer (PEMWE) [18]. In addition, Graeme J. Millar also constructed a thermally integrated electrolyzer, and the MD system employed waste heat from water electrolysis to provide high-quality water extracted from brine solutions [17]. These studies validate the potential benefits of integrating MD to remove excess heat producers during electrolysis processes, and the pure water obtained from MD can fully meet the water consumption in the electrolyzer.

In this work, the integrated processes of membrane distillation (MD) and water electrolysis are optimized by using a mathematical model to pinpoint the factors affecting the ratio of water production to water consumption, including electrolyzer power, seawater temperature, electrolyzer efficiency, and membrane distillation design parameters. Then, a technical and economic evaluation of the integrated technology is conducted to obtain the key factors affecting the cost of membrane distillation coupled with seawater desalination technology, and to promote its industrial application.

## 2. Methods

### 2.1. Coupled System Description

**Figure 1:**
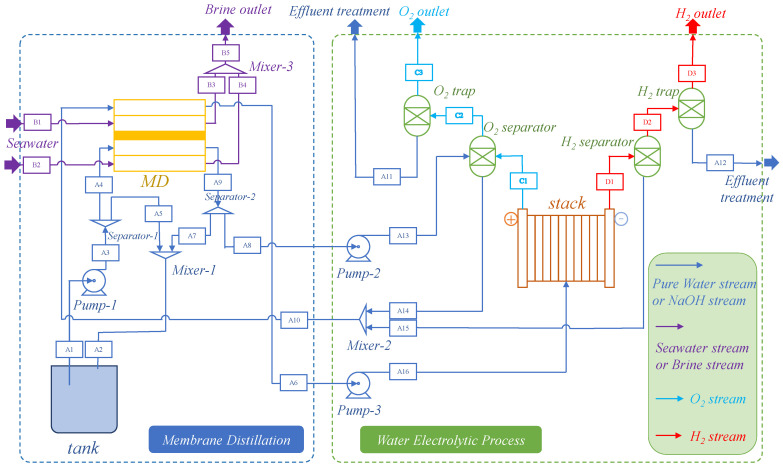
A flow diagram of the integrated seawater membrane distillation–water electrolysis system.

Figure 1 illustrates a flow diagram of the integrated seawater membrane distillation–water electrolysis system in this study. The system combines seawater membrane distillation (MD) technology with water electrolysis (WE) for hydrogen production, to conduct coupling and system optimization of material and energy flow.

For the MD process (the left part of Figure 1), seawater is introduced into the MD unit through a pump, and the waste heat from the water electrolysis unit is transferred to the seawater to heat the seawater. As the temperature rises, parts of the seawater begin to evaporate, generating water vapor. The water vapor is then further cooled by the evaporator, and condensed into pure water in the condenser. The generated pure water is discharged from the MD unit for electrolysis or storage. Meanwhile, the unevaporated seawater is gradually concentrated in the distillation process, forming a brine with a high salt concentration. The concentrated brine is discharged from the MD unit through a brine outlet. In purely theoretical calculations and assumptions made in simulations, heat losses due to heat dissipation in pipes and walls are neglected. In practice, heat dissipation can also be eliminated by reducing pipe lengths through the proximity of equipment and by using high-quality insulation materials. The flow in the MD unit is shown in Table 1.

In the right part of Figure 1, water splitting occurs in the electrolyzer. In the unit, water is introduced into the electrolyzer (stack), and then decomposed into hydrogen (H_2_) and oxygen (O_2_) through an electrochemical reaction. The generated hydrogen is first transported to the hydrogen trap (H_2_ trap) for preliminary collection, while oxygen is collected through the oxygen trap (O_2_ trap). After capture, hydrogen and oxygen enter specially designed separators for further purification and separation. Hydrogen is purified through the hydrogen separator (H_2_ separator) to remove unnecessary impurities, in order to obtain high-purity hydrogen. Similarly, oxygen also passes through the oxygen separator (O_2_ separator) to separate high-purity oxygen. Finally, pure hydrogen and oxygen are discharged from the system through their respective outlets, namely the H_2_ and O_2_ outlets, for subsequent storage or industrial applications. The flow in the electrolyzer unit is shown in Table 2.

### 2.2. Coupled System Simulation

**Figure 2:**
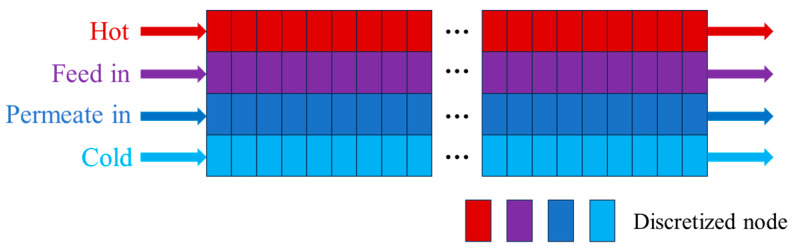
The finite element simulation of MD.

In the MD model, the hot flow (Hot), feed flow (Feed in), permeate flow (Permeate in), and cold flow (Cold) are treated independently. The MD unit is modeled using the finite element method to simulate the membrane distillation device’s heat and mass transfer process [19]. The entire device is divided into multiple small units, as shown in Figure 2, and each unit contains four different fluids. The hot flow enters from the top of the left side of the MD device, exchanges heat with other fluids while passing through the device, and finally discharges from the top of the right side. The feed flow is located in the second layer, and enters from the left side. After the heat exchange process, it is discharged to the right. The permeate flow is the steam passing through the membrane during the membrane distillation. It moves in the middle layer (third layer), and is finally discharged through the outlet. The cold flow is located at the bottom, and forms an effective energy exchange between the feed and hot flows through the fluid loop. That is, heat transfer occurs during the heating of seawater by the hot flow and the cooling of the infiltrating steam by the cold flow, while mass transfer is realized only through the evaporation of water from seawater to the infiltrating side.

The core goal of the electrolyzer model is to decompose water into hydrogen and oxygen through electrical energy, and then optimize and analyze the hydrogen production process. The electrolyzer model can simulate the electrolysis reaction of water, using an electric current to decompose water into hydrogen and oxygen. Here, an alkaline electrolytic cell is used for the simulation. The cathode reaction is 2H_2_O + 2e^−^ = H_2_ + 2OH^−^, and the anode reaction is 2OH^−^ = 0.5O_2_ + H_2_O + 2e^−^. Aspen Plus was used to build the model, and the model can calculate the hydrogen and oxygen production rates based on the feed, power, heat load, temperature, and pressure conditions [20,21]. The results of the MD module can be called in the electrolysis module to complete the simulation. The design parameters of the electrolyzer are shown in Table S1. In addition, the parameters of the membrane distillation modules and the composition of the simulated seawater are shown in Tables S2 and S3. The input parameters of the coupled system are listed in Table 3.

In the simulations, the following assumptions were used for the heat and mass transfer processes in MD [19]:

(1) In the direction perpendicular to the flow, the effects of temperature and concentration polarization are not considered separately, but are integrated in the experiments for the measurement of heat and mass transfer coefficients, respectively.
(2) A semi-infinite flat plate model is applied in the direction of heat and mass transfer, where temperature and flow are maintained constant.
(3) There are constant heat and mass transfer coefficients in the direction of the flow [22].
(4) The flow rate of the liquid stream is constant and does not vary with mass transfer.

### 2.3. Techno-Economic Analysis Method

**Figure 3:**
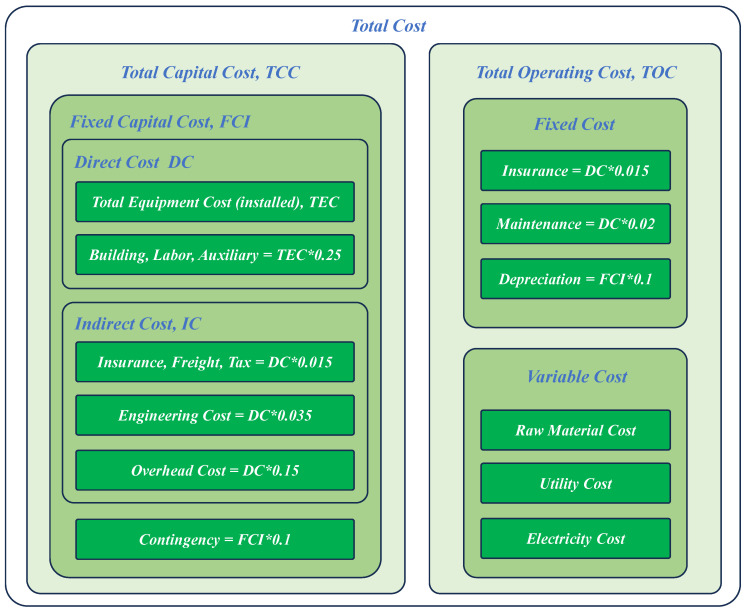
The framework of total cost.

In Figure 3, the Total Capital Cost can be divided into Fixed Capital Investment (FCI) and Contingency (10% of FCI). The FCI can be divided into Direct Cost (DC), including the Total Equipment Cost (TEC) and the Building, Labor, and Ancillary Costs (25% of TEC); and Indirect Cost (IC), including the Insurance, Freight, Tax (1.5% of DC), Engineering (3.5% of DC), and Overhead Costs (15% of DC). The Total Operation Cost can be divided into Fixed Cost (FC) and Variable Cost (VC). The FC contains Insurance (1.5% of DC), Maintenance (2% of DC), and Depreciation costs (10% of FCI), while the VC contains Raw Material, Utility, and Electricity costs.

Aspen Economic Analyzer was used to estimate equipment costs and make the following assumptions: 1. The Factory Cost Index (CEPCI) was used to adjust cost data for each year, and the CEPCI for 2021, 2022, and 2023 are 708.8, 699.0, 797.9, respectively. 2. The life of the alkaline water electrolysis plant is 20 years. 3. The utilization factor is 95%, equivalent to 8322 h of operation per year. 4. The land costs were ignored. US retail electricity prices are $0.105 per kWh in 2023. The electrolyzer costs $571 per kilowatt, and needs to be replaced every 10 years. The electrolyzer’s scale factor reflects its nonlinear cost change. The following Formula (1) was used to calculate the cost of an electrolyzer with different scales.

(1) $$\frac{C_{i}}{C_{j}}=(\frac{W_{i}}{W_{j}}{)}^{\alpha }$$

In the formula, *W_i_* and *W_j_* are the rated power of electrolyzers *i* and *j*, respectively. *C_i_* and *C_j_* are the costs of electrolyzers *i* and *j*, respectively. *α* is 0.85 for an alkaline water electrolyzer.

The levelized costs were calculated using the following Formulas (2) and (3) [23,24].

(2) $$LCOW\left(\$/ton\right)=\frac{\frac{TCC}{n}+TOC}{m_{PW}}-PW\times \frac{[i(1+i){]}^{n}}{(1+i{)}^{n-1}}$$

(3) $$LCOH\left(\$/ton\right)=\frac{\frac{TCC}{n}+TOC}{m_{H}}-H\times \frac{[i(1+i){]}^{n}}{(1+i{)}^{n-1}}$$

*LCOW* is the levelized cost of water, and *LCOH* is the levelized cost of hydrogen. *m_PW_* is the amount of pure water produced in MD annually, *m_H_* is the amount of hydrogen produced annually, *n* is the operating years of the plant (20 years), and *i* is the interest rate (4.5%).

### 2.4. Return on Investment Analysis Method

The calculation of return on investment (*ROI*) analysis was as follows (4) [21].

(4) $$ROI=\frac{H_{2}\;price\times H_{2}\;amount-TOC}{TCC}$$

The net income was calculated using the total hydrogen revenue generated by the project minus *TOC*. The revenue of pure water was ignored in this calculation.

## 3. Results and Discussion

### 3.1. Running Results

The parameters that were used to run the model are shown in Table 1, Table 2 and Table 3, and the results are shown in Table 4. The pure water production of MD is 51.36 kg/h, larger than the water consumption of STACK (49.65 kg/h). The amount of water the MD device generates is thoroughly utilized by the electrolyzer (Stack) to meet the water demand in the coupled system. At the same time, the waste heat generated during the electrolyzer’s operation is also successfully used to heat the feed seawater of the MD device, thus achieving effective recovery and utilization of heat energy. Such energy coupling reduces the system’s dependence on external energy and significantly improves the overall energy efficiency, making the system more environmentally friendly and economical.

### 3.2. Operating Parameter Analysis

The integrated system’s paramount goal is to produce enough water from the waste heat of electrolysis to support the water consumed by electrolysis. Hence, sensitivity analysis was used to evaluate the effect of several parameters, including electrolyzer power, seawater temperature, and MD parameters, on the water production/consumption ratio, in order to optimize the integrated system.

#### 3.2.1. Electrolyzer Power

Electrolyzer power is one of the most critical parameters in this integrated system. It directly determines the energy input of the electrolysis process, and impacts the system’s operating scale and output efficiency. The higher the power, the more water the electrolyzer can handle, and the higher the resulting hydrogen and oxygen production will be, increasing the system’s overall output.

**Figure 4:**
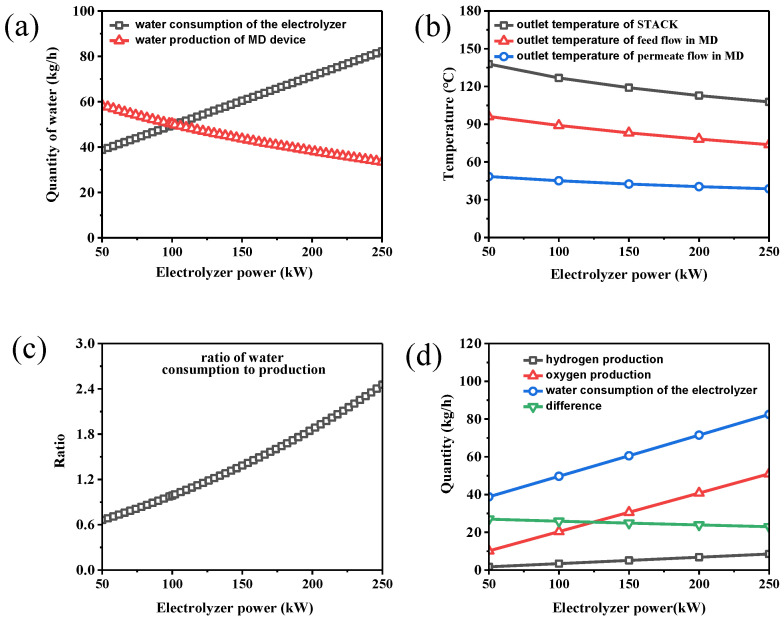
The effect of electrolyzer power on (**a**) water production and consumption, (**b**) outlet temperature, (**c**) ratio of water consumption/production, and (**d**) production of hydrogen and oxygen.

Figure 4a shows the relationship between the electrolyzer power (kW) and the water consumption of the electrolyzer (kg/h) or the water production of the MD device (kg/h). As the electrolyzer power increases, the water consumption increases accordingly. Figure 4b shows the relationship between the electrolyzer power and the temperature of each outlet stream. When the electrolyzer power remains constant, the heat load also remains stable. When the electrolyzer power increases, the water consumption of the electrolyzer increases, and the temperature change of the flow through the electrolyzer becomes small. That is, the outlet temperature of the electrolyzer stack becomes low. Then, the temperature difference to drive the mass transfer in MD desalination becomes smaller, leading to a decrease in the pure water production of MD. That is, as the power of the electrolyzer increases, the water production of the MD device gradually decreases, as shown in Figure 4a.

At the equilibrium point (the intersection of the two curves in Figure 4a), the electrolyzer’s water consumption matches MD’s water production, and the system can achieve a self-sufficient material balance. This means that the MD device fully utilizes the waste heat generated by the electrolyzer, and the water generated by the MD device just meets the water demand of the electrolyzer. The ratio of MD water production to water consumption of the electrolyzer was defined. As shown in Figure 4c, when the electrolyzer power reaches 102 kW, the ratio is about 100%. Furthermore, when the ratio exceeds 100%, this means that MD’s water production exceeds the electrolyzer’s water consumption. That is, this coupling device can be used to produce both hydrogen and pure water.

The water consumption for the production of hydrogen and oxygen is not precisely equal to the water consumption of the electrolyzer (Figure 4d). Hydrogen and oxygen streams can carry a certain amount of water vapor or liquid water, which cannot be returned to the electrolyzer for recycling. The water discharge is defined as the difference between the water consumption for electrolysis and the water consumption for hydrogen production. As the power increases, the water discharge gradually decreases. As the power increases, the outlet temperature decreases; thus, the water loss becomes smaller. A condenser can collect this water to further reduce the loss.

#### 3.2.2. Seawater Temperature

Due to environmental factors such as climate, seawater temperature fluctuates significantly with the seasons, directly impacting the integrated system’s performance. The seawater temperature affects the equipment’s heat transfer efficiency, and has a knock-on effect on the output of the individual streams.

**Figure 5:**
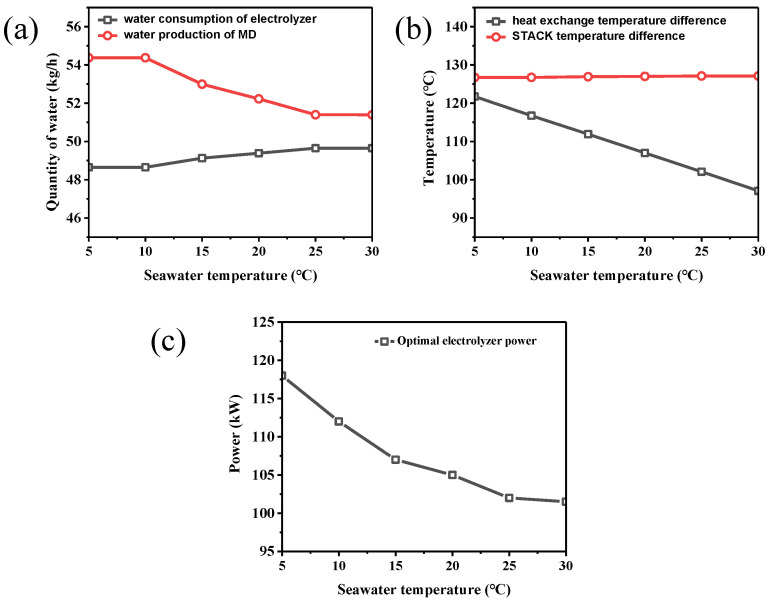
The effect of seawater temperature on (**a**) water production and consumption, (**b**) heat exchange/stack temperature difference, and (**c**) the equilibrium point between water production and consumption.

As shown in Figure 5a, an increase in seawater temperature improves the operating efficiency of the electrolyzer. This is because higher temperatures can effectively promote ion migration in the electrolyte solution, thereby accelerating the electrolysis reaction of water. In this case, the energy required by the electrolyzer can be reduced. In seawater with low temperatures, the ion conductivity in the electrolyte solution is small, thereby increasing the resistance of the solution. To maintain average electrolysis efficiency, the system needs to input more electrical energy, leading to a low working efficiency of the electrolyzer.

As shown in Figure 5b, the heat exchange temperature difference decreases as the seawater temperature increases. When the seawater temperature is high, the temperature difference between the hot stream and the seawater decreases, leading to a decrease in the driving force of mass transfer in the membrane distillation process. On the contrary, the low-temperature seawater helps to improve the mass transfer efficiency of the MD device, prompting the evaporation and condensation rates of water, resulting in an increase in the production of distilled water.

The change in seawater temperature can cause a shift in the equilibrium point between the water production in MD and the water consumption of the electrolyzer. As shown in Figure 5c, the optimal power rises rapidly as the seawater temperature gradually decreases. At lower seawater temperatures, the electrolyzer should maintain a higher power output to consume the pure water. At the same time, the increase in membrane distilled water production means that the system can effectively use more waste heat for water distillation and recovery, which further increases power demand. Therefore, the optimal operating point moves toward higher power as the seawater temperature decreases.

#### 3.2.3. MD Parameters

In the membrane distillation process, the parameters of the MD device can directly affect the heat and mass transfer efficiency, and, thus, the overall performance of the coupling device.

**Figure 6:**
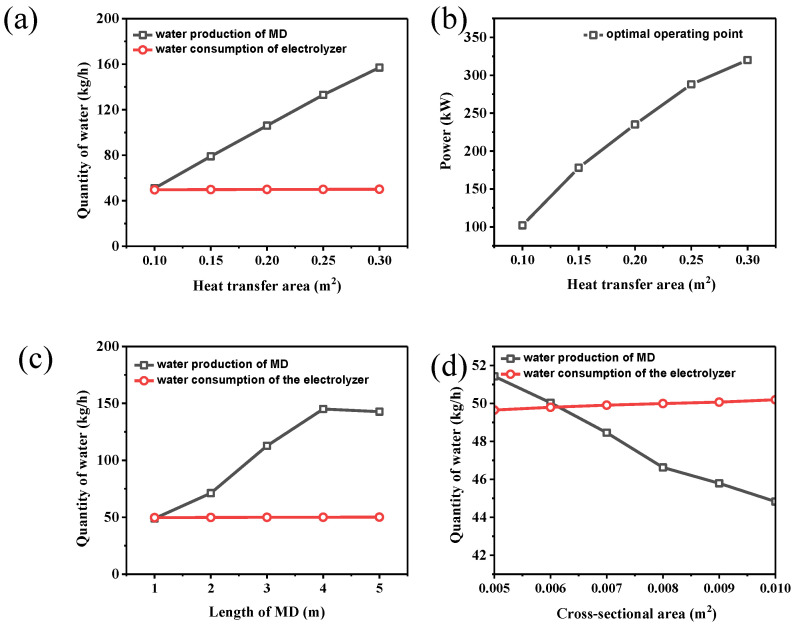
The effect of MD heat transfer area on (**a**) water production and consumption and (**b**) the equilibrium point between water production and consumption; the effect of the (**c**) length and (**d**) cross-sectional area on water production and consumption.

Figure 6a shows the effect of the heat transfer area of MD on the water production in MD and the water consumption of electrolyzed water. With an increase in the heat exchange area, the water production of membrane distillation shows an apparent linear growth trend.

When the heat exchange area is increased from 0.1 m^2^ to 0.3 m^2^, the water yield of membrane distillation rises from about 60 kg/h to nearly 160 kg/h. These results show that increasing the heat exchange area can significantly improve heat transfer efficiency in the membrane distillation process, promote the evaporation and condensation of water, and directly increase the water yield. The water consumption of electrolyzed water remains stable in the range of the heat exchange area, about 40 kg/h. The change in the heat exchange area has no noticeable effect on the consumption of electrolyzed water.

As shown in Figure 6b, the system’s productivity increases significantly as the MD heat transfer area increases. This improvement is mainly reflected in the expansion of the heat exchange area, which allows more heat to be effectively transferred and enhances the overall performance of the membrane distillation unit. To maintain the system’s optimal operating point in this situation, it is necessary to increase the power of the electrolyzer simultaneously to achieve a balanced coupling result of energy and material.

As shown in Figure 6c, the increase in the MD length significantly affects the membrane distillation’s water yield. In contrast, the water consumption of electrolyzed water remains stable over the whole range of heat exchange areas. As shown in Figure 6d, an increase in MD’s cross-sectional area (m^2^) reduces the water yield of membrane distillation. The flow rate is reduced due to the large cross-sectional area, which reduces the mass transfer efficiency and evaporation rate. The amount of water consumed by electrolysis increases slightly with the increase in the MD cross-sectional area. Overall, although increasing the cross-sectional area of MD may improve heat transfer, the amount of water generated by membrane distillation does not increase, but decreases, due to the limitations of flow rate and mass transfer efficiency. Therefore, in practical applications, the interaction between flow rate and heat and mass transfer efficiency must be considered when optimizing the cross-sectional area of MD to achieve the best water treatment effect.

The Specific Electrical Energy Consumption (SEEC, kWh/m^3^) and Specific Thermal Energy Consumption (STEC, kWh/m^3^) of the MD process were also calculated (as shown in Section S4 of the SI File) [25,26,27,28,29,30,31,32,33]. The results show that the SEEC and STEC are 881.21 kWh/m^3^ and 1.20 kWh/m^3^, respectively. Furthermore, the thermal efficiency η of MD is about 12.79%.

### 3.3. Techno-Economic Analysis

#### 3.3.1. Levelized Cost

**Figure 7:**
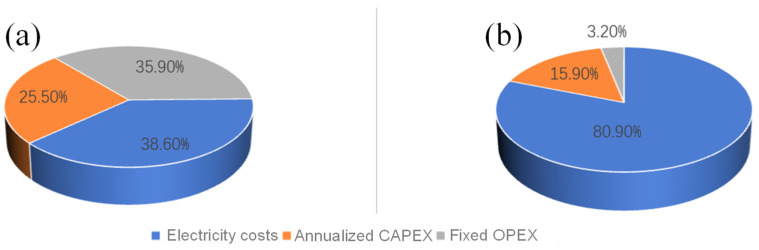
Economic analysis results: the costs of (**a**) membrane distillation for desalination and (**b**) electrolysis of water for hydrogen production.

Figure 7 shows the economic analysis results. The desalination process’s leveling cost (LCOW) is approximately US$1.28 per tonne of water (Figure 7a). Among its cost components, electricity costs (38.6%) dominate and are the main factor influencing the leveling cost, followed by fixed OPEX (Operational Expenditure) (35.9%) and annualized CAPEX (Capital Expenditure) (25.5%).

In Figure 7b, the cost of producing hydrogen through the alkaline water electrolysis process is $1.37/kg H_2_. This cost consists primarily of electricity costs (80.9%), followed by annualized CAPEX (15.9%) and fixed OPEX (3.2%).

In addition to the cost of electricity, annualized capital expenditures account for a large proportion, closely related to the initial investment in equipment and its depreciation calculation. Raw material costs, fixed operating costs, and maintenance and operating expenses are important factors affecting the total cost. Although these costs account for a relatively small proportion, they still play a vital role in the overall leveling costs, which have a combined impact on the economics and cost-effectiveness of desalinated water and hydrogen production.

#### 3.3.2. Parameter Analysis

**Figure 8:**
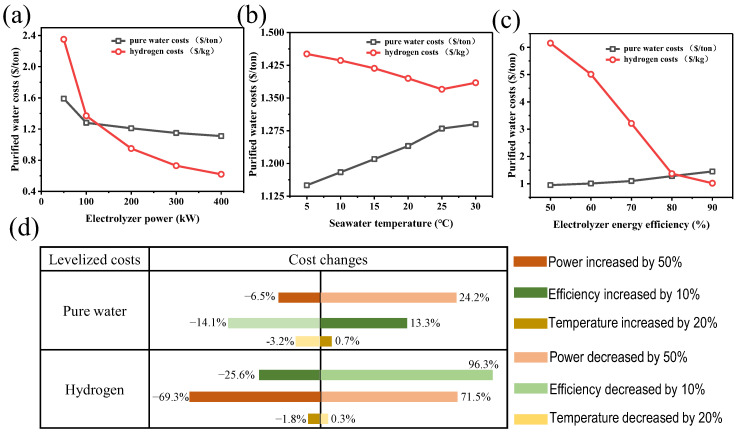
The effects of (**a**) electrolyzer power, (**b**) seawater temperature, and (**c**) electrolyzer energy efficiency on the levelized cost of pure water and hydrogen; (**d**) a tornado chart of the production cost.

Sensitivity analysis was used to investigate the impact of significant parameters on economic analysis. Three parameters, including electrolyzer power, were investigated here. As the electrolyzer power increases, the levelized cost of pure water and hydrogen gradually decreases (Figure 8a). With the rise in seawater temperature, the levelized cost of pure water gradually increases, while the price of hydrogen gradually decreases (Figure 8b). With the increase in electrolyzer energy efficiency, the levelized cost of pure water increases, while the average price of hydrogen decreases gradually (Figure 8c).

Figure 8d shows the levelized cost changes according to the parameters in a tornado chart. It can be seen that the most critical factor affecting the production cost of pure water is the electrolyzer power, followed by the electrolyzer energy efficiency and, finally, the seawater temperature. The levelized costs of pure water in the MD unit decrease by 6.5% with a 50% increase in electrolyzer power, increase by 13.3% with a 10% increase in electrolyzer energy efficiency, and increase by 0.7% with a 20% increase in seawater temperature. The most important factors affecting the hydrogen production cost are the electrolyzer energy efficiency, the electrolyzer power, and the seawater temperature. The levelized costs of hydrogen in the electrolyzer unit decrease by 25.6% with a 10% increase in electrolyzer energy efficiency, decrease by 69.3% with a 50% increase in electrolyzer power, and decrease by 1.8% with a 20% increase in seawater temperature.

#### 3.3.3. Return on Investment Analysis

**Figure 9:**
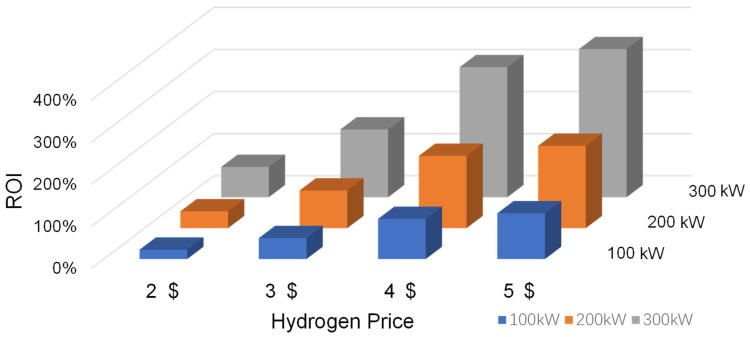
The impact of hydrogen prices and electrolyzer power on ROI.

Return on investment (ROI) measures the ratio of investment return to cost, and is a common indicator used to evaluate a project’s economic benefits and investment value [34,35]. The impact of hydrogen prices and electrolyzer power on ROI was assessed, as illustrated in Figure 9. The horizontal axis represents the hydrogen price ($/kg), the vertical axis represents the ROI (%), and the columnar bars of different colors correspond to electrolyzers of varying power classes (100 kW, 200 kW, 300 kW).

With the rise in hydrogen prices, ROI shows a significant growth trend. The ROI increases dramatically as hydrogen prices gradually increase from $2 to $5. Specifically, when hydrogen prices are low ($2), the ROI value is lower, and the economic benefits are not obvious. In contrast, when the price of hydrogen reaches $5, the ROI value rises sharply, even exceeding 350% at 300 kW power. These results suggest that hydrogen prices are a crucial factor affecting the economic benefits of the coupled system. Increasing the price of hydrogen can significantly increase ROI and make the project more economically attractive. Therefore, continuous monitoring of hydrogen market price changes and appropriate risk management measures are vital to ensuring the long-term economic viability of the coupled system.

At the same hydrogen price, electrolyzers with different powers show different ROI trends. The ROI also increases significantly as the electrolyzer power increases from 100 kW to 300 kW. The electrolyzer with a higher power shows a scale effect for improving economic efficiency. For example, when hydrogen is priced at $4, the ROI of a 100 kW electrolyzer is about 100%, while the ROI of a 300 kW electrolyzer is close to 250%. This trend suggests that higher-power electrolyzers can provide a higher return on investment under the same market conditions.

## 4. Conclusions and Outlooks

Using renewable-energy-driven seawater desalination and water electrolysis coupling processes to produce green hydrogen is an essential direction for the sustainable development of hydrogen energy. In this paper, a coupling model of a seawater membrane distillation–water electrolysis hydrogen production system was established, and sensitivity analysis of critical parameters, such as membrane area, flow rate, temperature, and electrolysis power, was carried out, alongside an economic and technical analysis of the system. To make the water yield of membrane distillation greater than the water consumption of electrolyzed water, it is necessary to reasonably optimize the design scheme so that the integrated system can show excellent adaptability and stability under different environmental conditions. An increase in the heat exchange area, an increase in the length of the heat exchanger tube, and a decrease in the cross-sectional area of the heat exchanger can significantly increase the water yield of the heat exchanger, but have little effect on the water consumption of the electrolyzer. The hydrogen market price is a crucial factor affecting the return on investment. When the price of hydrogen rises, the project’s revenue increases significantly. This effect can be mitigated by optimizing operational parameters, such as improving system efficiency and reducing energy consumption.

The integrated system offers excellent energy efficiency compared to conventional desalination and hydrogen production processes, and offers advantages in terms of environmental protection and resource conservation. In follow-up research, renewable energy must be integrated into the coupling system to study the impact of energy fluctuations on the integrated system of membrane distillation desalination and water electrolysis. Further exploration of how to reduce initial investment and operating costs and increase market competitiveness through large-scale production and advanced manufacturing technology is needed.

## Figures and Tables

**Table 1:** The flow in the MD unit.

Flow	From	To	Use
A1	Tank	Pump-1	Pure water for MD
A2	Mixer-1	Tank	Pure water to storage tank
A3	Pump-1	Separator-1	Pure water with pressure to Separator-1
A4	Separator-1	MD	Pure water to Separator-1
A5	Separator-1	Mixer-1	Extra pure water from Separator-1 to Mixer-1
A6	MD	Pump-3	Electrolyte after heat exchange to Pump-3
A7	Separator-2	Mixer-1	Extra pure water from Separator-2 to Mixer-1
A8	Separator-2	Pump-2	Pure water for electrolysis
A9	MD	Separator-2	Pure water produced by MD to Separator-2
A10	Mixer-2	MD	Electrolyte as heat source for MD
B1	Seawater inlet	MD	Seawater to MD as water source
B2	Seawater inlet	MD	Seawater to MD as cooling water
B3	MD	Mixer-3	Concentrated seawater from MD to Mixer-3
B4	MD	Mixer-3	Cooling water from MD to Mixer-3
B5	Mixer-3	Brine outlet	Concentrated seawater from Mixer-3 to discharge

**Table 2:** The flow in the electrolyzer.

Flow	From	To	Use
A11	O_2_ trap	Effluent treatment	Extra pure water from O_2_ trap to treatment
A12	H_2_ trap	Effluent treatment	Extra pure water from H_2_ trap to treatment
A13	Pump-2	O_2_ separator	Pure water from MD to electrolyzer
A14	O_2_ separator	Mixer-2	Hot electrolyte from O_2_ separator to Mixer-2
A15	H_2_ separator	Mixer-2	Hot electrolyte from H_2_ separator to Mixer-2
A16	Pump-3	Stack	Cool electrolyte to electrolyzer
C1	Stack	O_2_ separator	O_2_ flows into O_2_ separator with electrolyte
C2	O_2_ separator	O_2_ trap	O_2_ flows from O_2_ separator to O_2_ trap
C3	O_2_ trap	O_2_ outlet	O_2_ from O_2_ trap to outlet
D1	Stack	H_2_ separator	H_2_ flows into H_2_ separator with electrolyte
D2	H_2_ separator	H_2_ trap	H_2_ flows from H_2_ separator to H_2_ trap
D3	H_2_ trap	H_2_ outlet	H_2_ from H_2_ trap to outlet

**Table 3:** The input parameters of the coupled system.

	Initial Input Parameters	Values
STACK	Power	100 kW
	Feed ratio of cathode/anode	1:1
	Heat loading	20 kW
	Pressure	7 bar
SEP-O_2_/H_2_	Pressure	6.7 bar
	Heat loading	0 (adiabatic)
TRAP-O_2_/H_2_	Pressure	1 bar
	Temperature	25 °C
MD	Total length	1 m
	Divided nodes	500
	Heat transfer area	0.1 m^2^
	Cross-section area	0.05 m^2^
Seawater	Temperature	25 °C
	Salt (wt%)	3.5%

**Table 4:** The operation results of the simulation program for seawater membrane distillation coupled with alkaline water electrolysis for hydrogen production.

	Initial Input Parameters	Values
MD	Pure water production	51.36 kg/h
	Feed of hot electrolyte	405.43 kg/h
	Temperature of hot electrolyte	108.8 °C
	Feed of cool seawater	5000 kg/h
	Temperature of cool seawater	25 °C
	Feed of seawater for desalination	500 kg/h
STACK	Water consumption	49.65 kg/h
	Feed	405.43 kg/h
	Feed temperature	108.1 °C
	Outlet temperature	127.2 °C
	One-cell voltage (100 cells)	1.4811 W
	electric current	607.64 A
TRAP-O_2_	Oxygen production	20.369 kg/h
	Hydrogen production	3.404 kg/h

## Data Availability

The original contributions presented in this study are included in the article/Supplementary Materials. Further inquiries can be directed to the corresponding author(s).

## Supplementary Materials

The following supporting information can be downloaded at https://www.mdpi.com/article/10.3390/membranes15020060/s1. Table S1. Parameters of Electrolyzer. Table S2. Parameters of Membrane Distillation Modules. Table S3. Composition of simulated seawater. Table S4. Results of sensitivity and economic analyses. Table S5. Parameter values obtained from simulation. Table S6. Calculation results for STEC, SEEC and η.
