# Supplementary material for: Seawater Membrane Distillation Coupled with Alkaline Water Electrolysis for Hydrogen Production: Parameter Influence and Techno-Economic Analysis
Source: Membranes (Basel). 2025 Feb 11;15(2):60. doi: 10.3390/membranes15020060 (PMC11857186; doi:10.3390/membranes15020060)
Supplement: Supplementary file 1 [file membranes-15-00060-s001.zip › membranes-3343441-supplementary.pdf]

Supporting Information

Seawater Membrane Distillation Coupled with Alkaline Water Electrolysis for Hydrogen Production: Parameter Influence and Techno-Economic Analysis

Xiaonan Xu [1], Zhijie Zhao [2,3], Chunfeng Song [1,*], Li Xu [2] and Wen Zhang [2,3,*]

[1] Tianjin Key Laboratory of Indoor Air Environmental Quality Control, School of Environmental Science and Technology, Tianjin University, Tianjin 300350, China
[2] State Key Laboratory of Chemical Engineering, School of Chemical Engineering and Technology, Tianjin University, Tianjin 300350, China
[3] Tianjin Key Laboratory of Membrane Science & Desalination Technology, School of Chemical Engineering and Technology, Tianjin University, Tianjin 300350, China
* Correspondence: chunfeng.song@tju.edu.cn (C.S.); zhang_wen@tju.edu.cn (W.Z.)

S1. Electrolyzer

The relevant parameters of the membrane modules used in this study are shown in Table S1 below.

Table S1. Parameters of Electrolyzer.

Parameters	Value	
	cathode	anode
Activation area	5 m^2	5 m^2
Channel width	1 m	1 m
Channel length	5 m	5 m
Porosity	0.3	0.3
Curvature	3.8	3.8
Pore radius	1e-6 m	1e-6 m
Separation electrode and separator	1 m	1 m
Channel thickness	0.5 m	0.5 m
Electrode Roughness Factor	1.25	1.25
Porous material thickness	0.2 m	0.2 m
Bubble zone width	0.8 m	0.8 m
Channel roughness factor	1	1
Activation free energy	19229.48 kJ/kmol	12462.98 kJ/kmol
Reference exchange current density	1e-09 A/cm^2	0.001 A/cm^2
Resistivity	9.56e-08 Ωcm	9.56e-08 Ωcm
Charge transfer coefficient	1.65	0.73
Membrane activation area	2.5 m^2	
Membrane thickness	0.01 m	
Membrane porosity	0.42	
Membrane curvature	2.18	
Membrane humidity factor	0.85	
Membrane Oxygen Diffusivity	1.81e-05 cm^3/s	
Membrane hydrogen diffusivity	5.63e-05 cm^3/s	
Recombination reaction rate constant	1e10 kmol/m·h	
Permeation resistance factor	6	
Bruggeman's index	1.5	

S2. MD Modules

The parameters of membrane distillation modules are listed in Table S2, according to the experiments using a square cell with PTFE membranes, and obtained the relationships between heat and mass transfer coefficients and flow rates[19].

Table S2. Parameters of Membrane Distillation Modules.

Parameters	Value
Component Type	Flat-sheet
Material	PTFE
Manufacturer	Ningbo Chang Qi Fluoride Plastic Products Co., Ltd.
Pore size	0.5 μm
Water Contact Angles	140°± 5
Total length	1 m
Heat transfer area	0.1 m^2
Cross-section area	0.05 m^2

As above, the temperature and composition of the simulated seawater have an important effect on heat and mass transfer, and NaCl solution was used instead of seawater in this study (Table S3).

Table S3. Composition of simulated seawater.

Parameters	Value
Temperature	25 ℃
Salinity (NaCl wt%)	3.5%

S3. Sensitivity and economic analyses

Table S4. Results of sensitivity and economic analyses.

	Module	Parameters	Value	
LCOW	MD	Electricity cost	38.6%	
		CAPEX	25.5%	
		OPEX	35.9%	
	Electrolytic cell	Electricity cost	80.9%	
		CAPEX	15.9%	
		OPEX	3.2%	
Parameter analysis	Pure water	Electrolyzer power	+50%	-6.5%
		Electrolyzer energy efficiency	+10%	13.3%
		Seawater temperature	+20%	0.7%
	Hydrogen	Electrolyzer power	+50%	-69.3%
		Electrolyzer energy efficiency	+10%	-25.6%
		Seawater temperature	+20%	-1.8%

S4. Energy and efficiency

The thermal and electrical consumption and energy efficiency of the membrane distillation unit were evaluated according to the literature[25,26].

The Specific Thermal Energy Consumption (STEC, kWh/m^3) is the thermal energy consumption to produce 1 m^3 of produced water, that is the ratio of the heat consumed to warm up the feed flow to the flow rate of the product (permeate channel), which is given by the following equation S1.

$$STEC = \frac{m_f \cdot C_{p,f} \cdot (t_{hi} - t_{co}) \cdot \rho_w}{3.6 \times 10^6 \cdot m_p} \qquad (S1)$$

In the equation, m_f and m_p are the mass flow rate of feed and permeate flow, kg/h, respectively. ρ_w is the density of the produced water, kg/m^3. $C_{p,f}$ is the specific heat capacity of the feed, J/(kg·℃). t_{hi} and t_{co} are the inlet temperature of the hot flow and the outlet temperature of the cold flow, ℃, respectively.

The Specific Electrical Energy Consumption (SEEC, kWh/m^3) is the electrical energy consumption of the hot, feed and cold flow pumps produced per unit volume of permeate, and the equation is as S2.

$$SEEC = SEEC_h + SEEC_c + SEEC_f$$
$$= \frac{1}{36000}\left[\left(\frac{\Delta p_h}{\eta_h}\right)\cdot\left(\frac{m_h}{m_p}\right) + \left(\frac{\Delta p_c}{\eta_c}\right)\cdot\left(\frac{m_c}{m_p}\right) + \left(\frac{\Delta p_f}{\eta_f}\right)\cdot\left(\frac{m_f}{m_p}\cdot\frac{\rho_w}{\rho_f}\right)\right] \qquad (S2)$$

In the equation, m_c are the mass flow rate of cold flow, kg/h. Δp_h, Δp_c and Δp_f are the pressure drop of hot, cold and feed flow, mbar, respectively. η_h, η_c and η_f are the pump performance of the hot, cold and feed flow, respectively. ρ_f is the density of the feed flow, kg/m^3.

Thermal efficiency η can be defined as the ratio of thermal energy utilized (useful heat) to the total thermal energy input. The heat input comes from the hot flow, it contains the direct heat transfer in addition to the heat released by the phase change. And the useful heat can be calculated from the latent heat of the produced water, and then η can be calculated as following formula S3.

$$\eta = \frac{Q_c}{Q_i} \cdot 100\% = \frac{m_p \cdot h_{fg}}{m_h \cdot C_{p,h} \cdot (t_{hi} - t_{ho}) + m_h \cdot \Delta H_h} \cdot 100\% \qquad (S3)$$

In the equation, Q_c and Q_i are the heat consumed by the device and heat input to the device, kJ. h_{fg} is the latent heat of evaporation of water, kJ/kg. m_h is the flow rate of hot flow, kg/h. $C_{p,h}$ is the specific heat capacity of the hot flow, J/(kg·℃). t_{ho} are the outlet temperature of the hot flow, ℃. ΔH_h is the enthalpy of phase transition of hot flow, kJ/kg.

The values of the above mentioned parameters are listed in the following table S5.

Table S5. Parameter values obtained from simulation

Parameters	Value	Parameters	Value
m_f	500.00 kg/h	η_c	0.8[a]
m_p	51.36 kg/h	η_f	0.8[a]
m_c	5000.00 kg/h	η_h	0.8[a]
m_h	405.43 kg/h	Δp_c	300 mbar
ρ_w	1.00×10^3 kg/m^3	Δp_f	300 mbar
ρ_f	1.03×10^3 kg/m^3	Δp_h	300 mbar
$C_{p,f}$	3.89×10^3 J/(kg·℃)	t_{hi}	108.80 ℃
$C_{p,h}$	2.30×10^3 J/(kg·℃)	t_{ho}	104.05 ℃
h_{fg}	2260.00 kJ/kg	t_{co}	25.03 ℃
ΔH_h	2228.00 kJ/kg		

[a]values from literature[27]: Energy Conversion and Management 185(2019):143-154.

Based on the above equations and simulation result data, STEC, SEEC and η can be calculated as shown in Table S6 below.

Table S6. Calculation results for *STEC*, *SEEC* and η

Parameters	*STEC*	*SEEC*	η
Value	881.21 kWh/m^3	1.20 kWh/m^3	12.79%

The calculated values of *STEC*, *SEEC*, and η are closer to other literature related to membrane distillation[28-33]. Therefore, it can be considered that the membrane distillation process designed in this project is reasonable.

Reference

19. Hausmann, A.; Sanciolo, P.; Vasiljevic, T.; Weeks, M.; Duke, M. Integration of membrane distillation into heat paths of industrial processes. *Chem. Eng. J.* **2012**, *211-212*, 378-387, doi:https://doi.org/10.1016/j.cej.2012.09.092.
25. Requena, I.; Andrés-Mañas, J.A.; Zaragoza, G. Influence of internal design on the performance of pilot vacuum-assisted air-gap membrane distillation modules for brine concentration with solar energy. *Desalination* **2024**, *573*, 117218, doi:https://doi.org/10.1016/j.desal.2023.117218.
26. Hardikar, M.; Marquez, I.; Phakdon, T.; Sáez, A.E.; Achilli, A. Scale-up of membrane distillation systems using bench-scale data. *Desalination* **2022**, *530*, 115654, doi:https://doi.org/10.1016/j.desal.2022.115654.
27. Miladi, R.; Frikha, N.; Kheiri, A.; Gabsi, S. Energetic performance analysis of seawater desalination with a solar membrane distillation. *Energy Convers Manage* **2019**, *185*, 143-154, doi:https://doi.org/10.1016/j.enconman.2019.02.011.
28. Alawad, S.M.; Lawal, D.U.; Khalifa, A.E.; Aljundi, I.H.; Antar, M.A.; Baroud, T.N. Analysis of water gap membrane distillation process with an internal gap circulation propeller. *Desalination* **2023**, *551*, 116379, doi:https://doi.org/10.1016/j.desal.2023.116379.
29. Mahmoudi, F.; Moazami Goodarzi, G.; Dehghani, S.; Akbarzadeh, A. Experimental and theoretical study of a lab scale permeate gap membrane distillation setup for desalination. *Desalination* **2017**, *419*, 197-210, doi:https://doi.org/10.1016/j.desal.2017.06.013.
30. Cipollina, A.; Di Sparti, M.G.; Tamburini, A.; Micale, G. Development of a Membrane Distillation module for solar energy seawater desalination. *Chem Eng Res Des* **2012**, *90*, 2101-2121, doi:https://doi.org/10.1016/j.cherd.2012.05.021.
31. Ma, Q.; Xu, Z.; Wang, R. Distributed solar desalination by membrane distillation: current status and future perspectives. *Water Res* **2021**, *198*, 117154, doi:https://doi.org/10.1016/j.watres.2021.117154.
32. Vanneste, J.; Bush, J.A.; Hickenbottom, K.L.; Marks, C.A.; Jassby, D.; Turchi, C.S.; Cath, T.Y. Novel thermal efficiency-based model for determination of thermal conductivity of membrane distillation membranes. *J. Membr. Sci.* **2018**, *548*, 298-308, doi:https://doi.org/10.1016/j.memsci.2017.11.028.
33. Zhang, Y.; Peng, Y.; Ji, S.; Li, Z.; Chen, P. Review of thermal efficiency and heat recycling in membrane distillation processes. *Desalination* **2015**, *367*, 223-239, doi:https://doi.org/10.1016/j.desal.2015.04.013.
